# The blue mussel inside: 3D visualization and description of the vascular-related anatomy of *Mytilus edulis* to unravel hemolymph extraction

**DOI:** 10.1038/s41598-020-62933-9

**Published:** 2020-04-21

**Authors:** Mieke Eggermont, Pieter Cornillie, Manuel Dierick, Dominique Adriaens, Nancy Nevejan, Peter Bossier, Wim Van den Broeck, Patrick Sorgeloos, Tom Defoirdt, Annelies Maria Declercq

**Affiliations:** 1 Laboratory of Aquaculture and Artemia Reference Center, Faculty of Bioscience Engineering, Ghent University, Coupure Links 653, 9000 Ghent, Belgium; 2 Department of Morphology, Faculty of Veterinary Medicine, Ghent University, Salisburylaan 133, 9820 Merelbeke, Belgium; 3 Centre for X-ray Tomography (UGCT), Department Physics and Astronomy, Proeftuinstraat 86/N12, 9000 Gent, Belgium; 4 Research Group Evolutionary Morphology of Vertebrates, Ghent University, K.L. Ledeganckstraat 35, 9000 Ghent, Belgium; 5 Department of Pathology, Bacteriology and Poultry Diseases, Faculty of Veterinary Medicine, Ghent University, Salisburylaan 133, 9820 Merelbeke, Belgium; 6 Present Address: XRE nv. Bollebergen 2B box 1, 9052 Ghent, Belgium

**Keywords:** Computational biology and bioinformatics, Structural biology

## Abstract

The blue mussel *Mytilus edulis* is an intensely studied bivalve in biomonitoring programs worldwide. The lack of detailed descriptions of hemolymph-withdrawal protocols, particularly with regard to the place from where hemolymph could be perfused from, raises questions regarding the exact composition of aspirated hemolymph and does not exclude the possibility of contamination with other body-fluids. This study demonstrates the use of high resolution X-ray computed tomography and histology combined with 3D-reconstruction using AMIRA-software to visualize some important vascular-related anatomic structures of *Mytilus edulis*. Based on these images, different hemolymph extraction sites used in bivalve research were visualized and described, leading to new insights into hemolymph collection. Results show that hemolymph withdrawn from the posterior adductor muscle could be extracted from small spaces and fissures between the muscle fibers that are connected to at least one hemolymph supplying artery, more specifically the left posterior gastro-intestinal artery. Furthermore, 3D-reconstructions indicate that puncturing hemolymph from the pericard, anterior aorta, atria and ventricle in a non-invasive way should be possible. Hemolymph withdrawal from the heart is less straightforward and more prone to contamination from the pallial cavity. This study resulted simultaneously in a detailed description and visualization of the vascular-related anatomy of *Mytilus edulis*.

## Introduction

Bivalve hemolymph is extensively used in a range of research domains such as ecotoxicology^1^, ecophysiology^2^ and (immune)toxicology^3^. *Mytilus edulis* (*M. edulis* Linnaeus, RRID:SCR_006069) hemolymph is also widely used in bio-accumulation and environmental studies concerning heavy metals such as cupper, mercury and cadmium^4–6^. Mussels are sessile filter-feeders: they continuously filter the surrounding water, making them excellent model organisms for eco-toxicological and eco-physiological studies. For example, studies on the uptake, fate and biological consequences of ingested microplastics^2,7^, toxicity assays with heavy metals^1^ and immunotoxic research^3^ all depend on the collection of hemolymph. Depending on the nature of the research, hemolymph extraction techniques vary from invasive (lethal) to non-invasive procedures and from single to multiple withdrawals. The main hemolymph collection site in bivalves is the adductor muscle (anterior or posterior, depending on the species) and less frequently the ventricle of the heart (Table 1). The pericardium and the extrapallial cavity are other puncture sites reported for harvesting body fluids^8,9^.

**Table 1:** Overview of different circulatory fluid collection sites, extraction methods, extracted volumes and the needle gauges.

Puncture site	Procedure	Species	Reference
Posterior adductor muscle	NI: Hole in shell; 26G needle; multiple sampling; 0.5–1 mL	*Rangia cuneata*	(Fyhn & Costlow, 1975)^8^
	NI: Notch in shell; 23G needle; multiple sampling; 200–5500 µL	*Crassostrea virginica*	(Ford, 1986)^50^
	NI: Puncture hinge joint; 22G needle; 50–200 µL	*Mytilus trossulus*	(Yanick & Heath, 2000)^51^
	NI: Notch in shell with plier; 21G needle	*Saccostrea glomerata*	(Moreira, Browne & Coleman, 2013)^52^
	NI: Hole in shell with dremel; 21G needle	*Saccostrea glomerata*	(Moreira, Browne & Coleman, 2013)^52^
	NI: Notch in shell; 21 G needle	*Mytilus edulis*	(Al-Subiai, Jha, & Moody 2009)^53^
	NI: Prise shells apart with knife; 21G needle	*Mytilus edulis*	(Al-Subiai, Jha, & Moody 2009)^53^
Anterior adductor muscle	NI: Prise shells apart; 25G needle; multiple sampling; 300–1500 µL	*Elliptio complanata*	(Gustafson *et al*., 2005)^27^
Ventricle	I: Removal of shell; glass capillary; 30–200 µL	*Rangia cuneata*	(Fyhn & Costlow, 1975)^8^
	I: Removal of shell; dissecting microscope; 25G needle	*Elliptio complanata*	(Gustafson *et al*., 2005)^27^
Pericard	NI: Puncture hinge joint; 26G needle; 500–1000 µL	*Rangia cuneata*	(Fyhn & Costlow, 1975)^8^
	NI: Hole in right shell; 16G needle	*Crassostrea virginica*	(Friedl, Alvarez, Johnson & Gratzner., 1988)^11^
Extra pallial space	NI: Needle	*Mytilus galloprovincialis*	(Calvo-Iglesias *et al*., 2016)^54^
	NI: Through mantle tissue; no adequate volume	*Elliptio complanata*	(Gustafson *et al*., 2005)^27^
	NI: Between shell and mantle; 8 cm needle	*Mytilus edulis*	(Zittier, Bock, Lannig & Portner, 2015)^9^
	NI: Between shell and mantle; needle	*Mytilus edulis*	(Thomsen *et al*., 2010)^37^
	NI: Hole in shell with round dental burr; cemented glass capillary	*Mytilus edulis* *Mercenaria mercenaria* *Crassostrea virginica*	(Crenshaw, 1972)^36^

Abbreviations: I: invasive technique which is often lethal; NI: non-invasive technique (if performed correctly) not lethal and multiple sampling in time possible; G: gauges; mL: milliliters; µL: microliters; cm: centimeters.

In bivalves the term hemolymph refers to the colorless blood, constituted of hemocytes (blood cells) and plasma (cell free hemolymph). The blood volume of bivalves is large. According to Martin, Harrison, Huston & Stewart^10^ the blood volume of *Mytilus californianus* is 50.8% of the wet body weight excluding the shell, ranging from 21.0–60.6 mL for mussels between 38.8 and 122.4 g, respectively. In contrast to vertebrates, bivalves have an open cardiovascular system that is not rigidly enclosed^11^. This makes it difficult to distinguish whether a structure in which hemolymph, or an ultra-filtrate^12^, or even only mere sea water is punctured. Circulating hemocytes are of vital importance in many biological processes, especially in immunological responses^13–16^. In mussels, the hemocytes are also involved in the host immune system as half of the genes expressed by these cells are antimicrobial peptides such as myticins, mytilins and myticilins^17^. Myticin C for example inhibits the replication of bacteria^18^, fish viruses^19^, and human herpesvirus^20^. In adult oysters, the gill was reported to be the potential hematopoietic site^21,22^. Hematopoietic development would be conserved across different species^23–25^. However, in bivalves, the origin of hematopoietic tissues and hemocytes deserves in-depth research. Hemocytes are able to actively migrate throughout the bivalve’s body. Thus, individual cells will be encountered at different places and at different times. Also the number of hemocytes in the hemolymph varies across taxa, among individuals of the same species and even within a single individual depending on its physiological state^26^. Gustafson *et al*.^27^ noted that cell counts and calcium levels differed significantly between hemolymph collected from the ventricle of the heart and from the adductor muscle. They attributed this to the sequence in sampling (first the adductor muscle), but further information to confirm this hypothesis was not provided in the study^27^. Picken^12^ mentioned another difference between pericardial and ventricular fluid composition. Contraction of the atria fills the ventricle with hemolymph but also forces some fluid through the walls of the atria into the pericardial cavity. The podocytes surround the outer surface of the atria form the pericardial gland, which ultra filtrates the hemolymph^26^. Pericardial content is therefore also called ultra-filtrate and its composition is somewhat different from that of ventricular hemolymph. The pericardial fluid is generally isotonic with the blood but contains less non-mineral substances (assumed proteins)^12^. Hence, when using hemolymph, it is important to accurately know where to sample. However, when puncturing the heart, it is difficult to distinguish whether the obtained fluid originated from the pericardial cavity or from the ventricle^27^. Furthermore, when sampling hemolymph from the most commonly used extraction site – the adductor muscle – it is not clear where exactly the obtained fluid comes from. Based on histologic research in the giant clam *Tridacna gigas*, Norton & Jones^28^ referred to hemolymph sinuses situated in between the microscopic muscle fibers, whereas other authors suggested the existence of a large adductor sinus^29^. But only few authors describe an actual vessel providing/draining hemolymph towards/from the adductor muscle^29,30^.

Despite the extent by which hemolymph is used in all kinds of assays, few histological^28^ or anatomical reconstructive images are available in literature to visualize and reconstruct the internal anatomy and cardiovascular system of bivalves.

This study demonstrates the use of high resolution X-ray computed tomography (micro-CT) and histology combined with 3D-reconstruction using AMIRA-software to visualize, help locating and understand the anatomy and physiology of bivalves.

## Results

### Anatomic orientation

**Figure 1:**
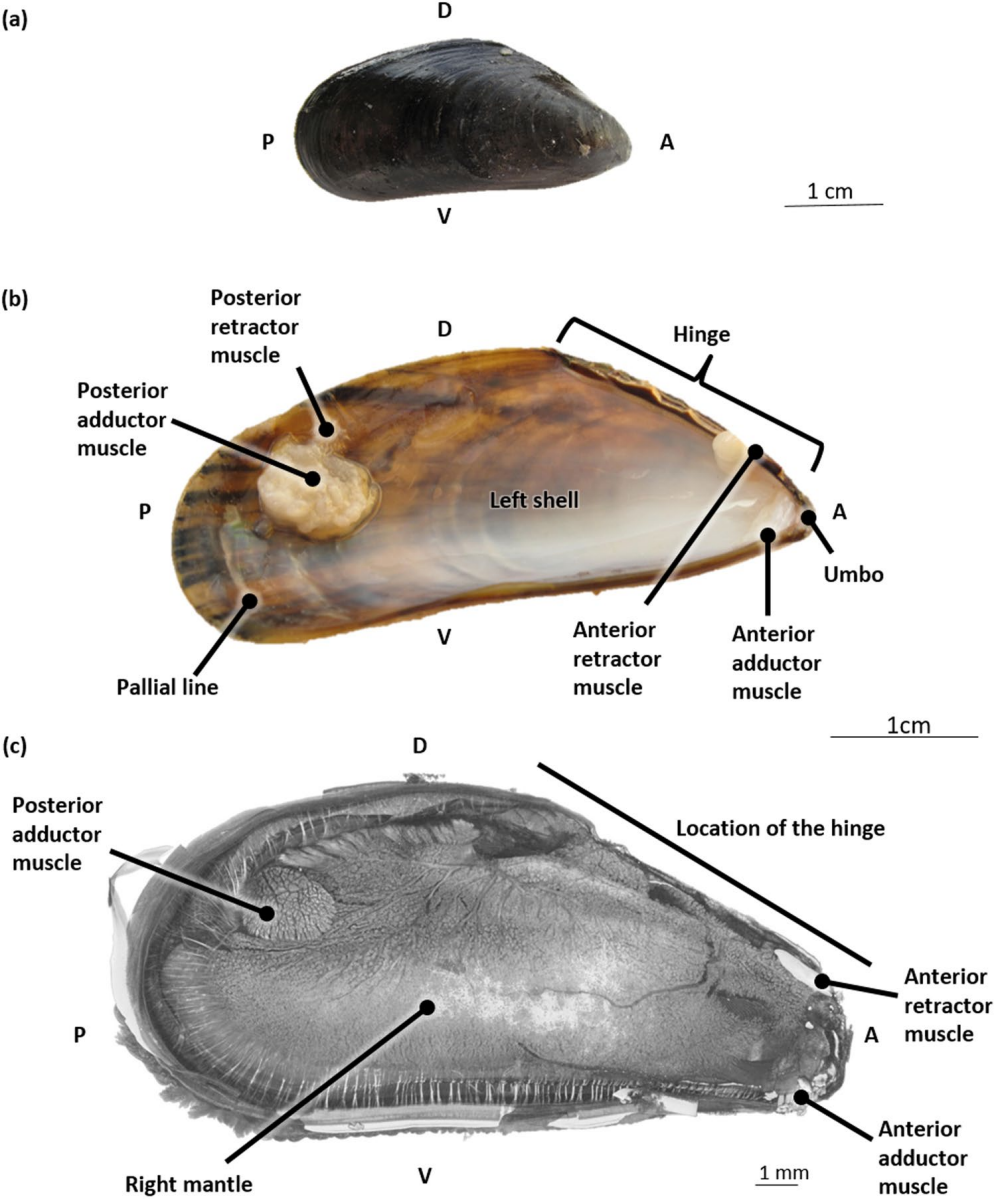
An overview of the most important terminology needed for orientation of the blue mussel (*Mytilus edulis*). (**a**) The outside of the right shell. (**b**) The inside of the left shell. (**c**) Without shells, right lateral overview generated from a micro-CT scan. Abbreviations: D, Dorsal; V, Ventral; A, Anterior; P, Posterior.

**Figure 2:**
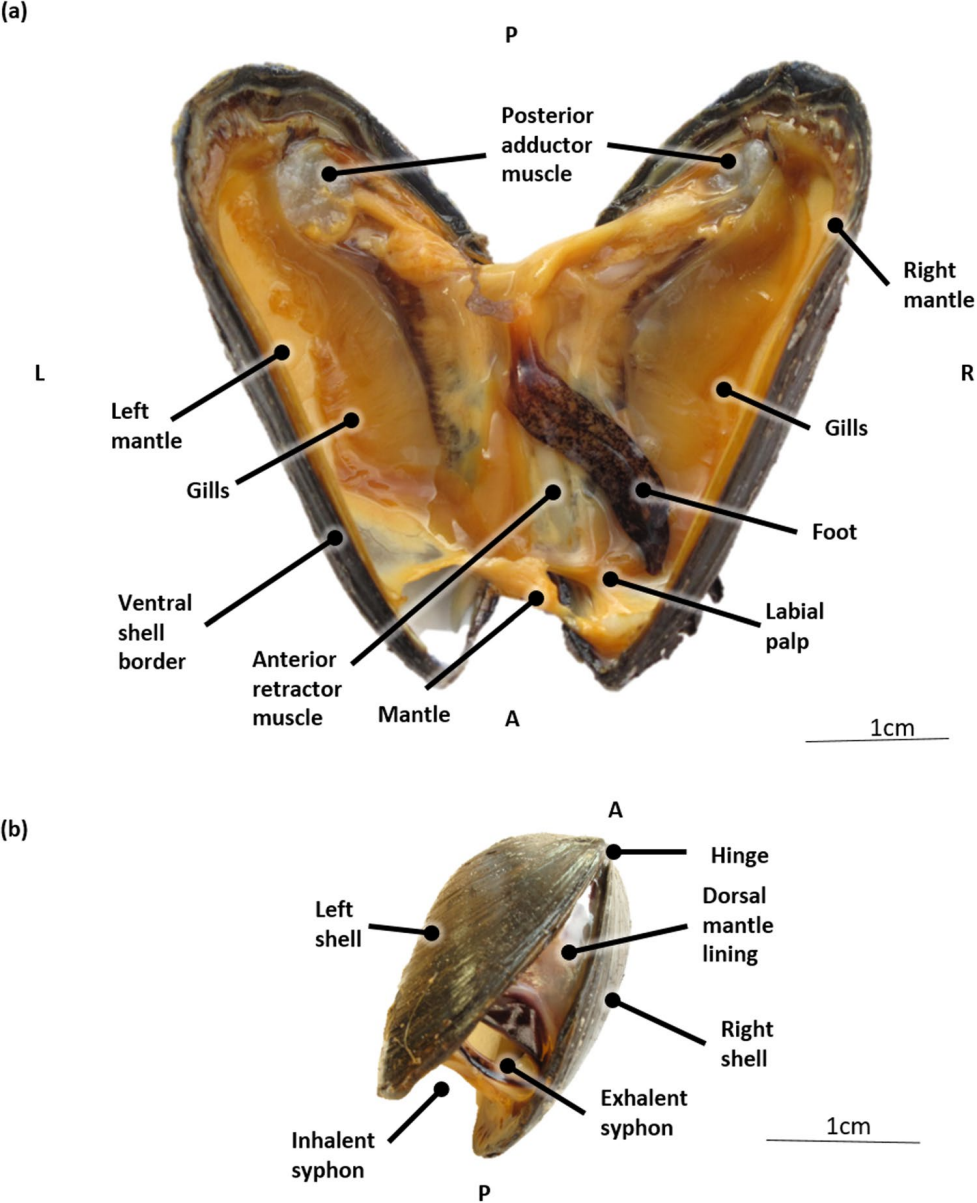
General anatomy of the blue mussel (*Mytilus edulis*) tissues. (**a**) Ventral view after cutting the adductor muscles and forcing the valves to open, hereby rupturing the connecting mantle parts. (**b**) Oblique dorsal view on a sedated (MgCl_2_) specimen. Abbreviations: L, Left; R, Right; P, Posterior; A, Anterior.

Figures 1 and 2 illustrate the general anatomy of *M. edulis* explaining some of the most important terms needed for orientation of the blue mussel throughout the rest of this manuscript. These figures are based on both macroscopic visualization of the mussel and its shell and on a micro-CT-scan generated in this study.

### 3D-visualization based on histological section images

**Figure 3:**
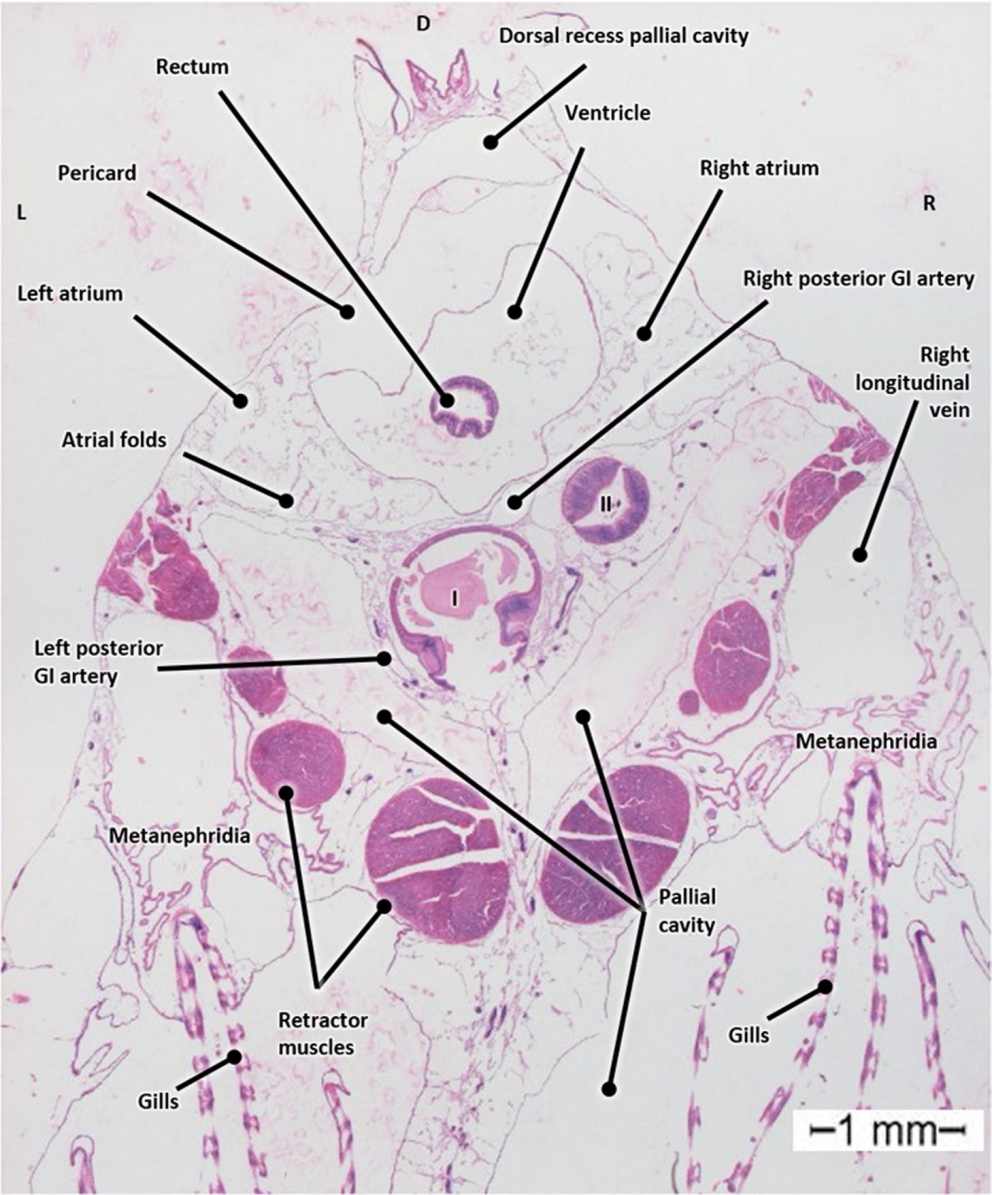
Transverse histological section of a Bouin fixated, H&E stained mussel (*Mytilus edulis*) at the level of the heart. Abbreviations: I, first gastro-intestinal segment; II, second gastro-intestinal segment; GI, Gastro-intestinal; D, Dorsal; L, Left; R, Right.

Fixation of mussels in a phosphate buffered 4% formaldehyde solution resulted in fragile tissue that easily fell apart during microtome sectioning. Light microscopic investigation of the H&E stained histological slides showed alteration of the internal morphology such as collapse of the intraluminal spaces. Best results were obtained with Bouin-fixative, as can be observed in Fig. 3, and was hence used as fixation fluid for further histologic interpretation of the mussel anatomy.

A 3D-reconstruction of histological serial sections visualizes the cardiovascular system (heart and related blood vessels), gastro-intestinal (GI) tract, gills, as well as the metanephridia, and the adductor and retractor muscles in relation to the foot (Supplementary Movie 1). This technique, although time-consuming, made it possible to evaluate the respective organ architecture and their relative position and relation to one another.

### Micro-CT

As for the results of the micro-CT visualization, the best contrast of the soft tissues was obtained by critical point drying. It was difficult to differentiate the heart with phosphomolybdic acid (PMA) staining (Supplementary Fig. S1) and injection of a contrast agent was not possible.

**Figure 4:**
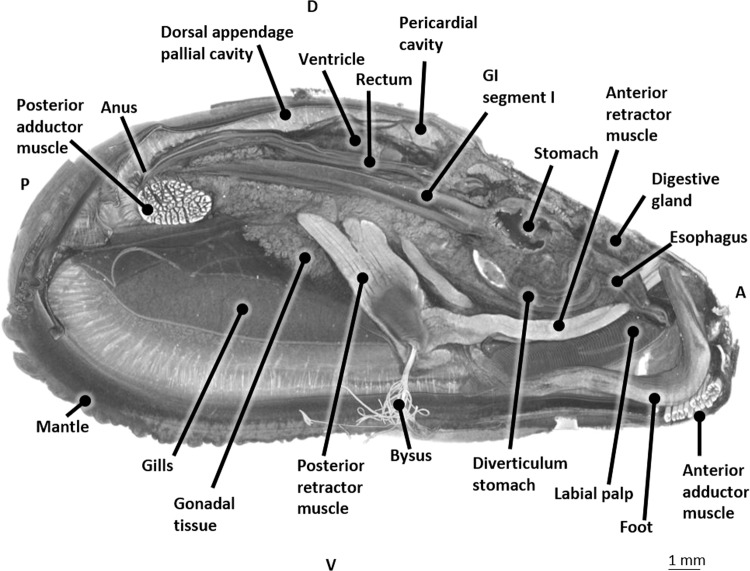
Longitudinal micro-CT section in 3D at the level of the heart of a critical point dried blue mussel (*Mytilus edulis*) after Bouin fixation. Abbreviations: GI, Gastro-intestinal; D,Dorsal; V, Ventral; P, Posterior; A, Anterior.

Critical point drying after Bouin fixation resulted in overall excellent contrast between different tissues and organs (Fig. 4). The ventricle and pericard were clearly distinguishable. These latter structures are pierced by the rectum. The location of the digestive gland along the longitudinal axis could be inspected extending from the mouth to the posterior adductor muscle and could be differentiated well from the GI-tract. Moreover, the mantle, musculature, and foot could be discriminated easily.

Mussels that filtered PMA with their shells forced open were better stained with PMA than the anesthetized mussels. The latter method was therefore abandoned. The obtained contrast of the soft tissues was acceptable; however, the cardiovascular system was hardly identifiable (Supplementary Fig. S1). The posterior adductor though had a denser structure and smaller interspace between the muscle fibers when stained with PMA in comparison to the critical point dried mussel in Fig. 4.

### Description of the anatomy of *Mytilus edulis* based on micro-CT imaging, histology and 3D-visualization

#### Shell, mantle and pallial cavity

**Figure 5:**
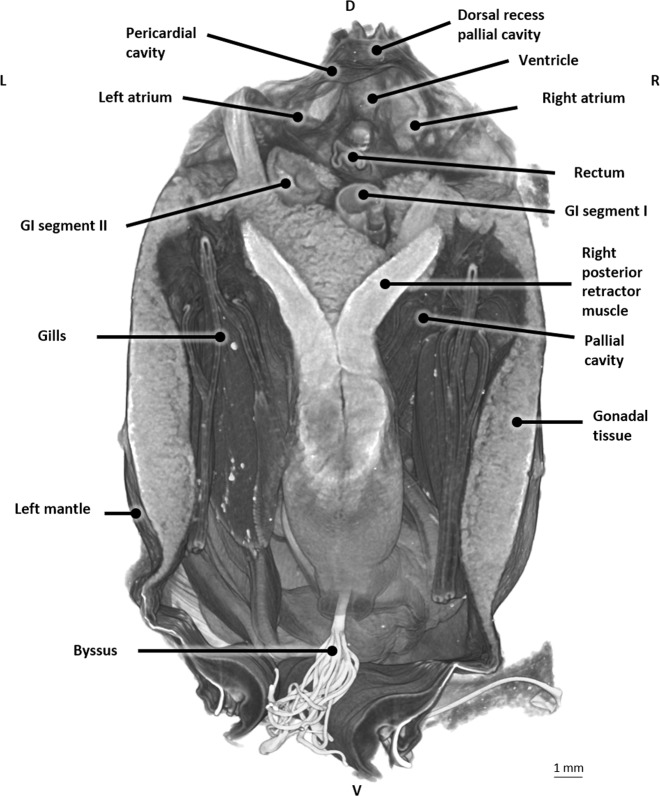
Transverse micro-CT section in 3D at the level of the heart of a critical point dried blue mussel (*Mytilus edulis*) after Bouin fixation. Abbreviations: GI, Gastro-intestinal; D, Dorsal; V, Ventral; L, Left; R, Right.

The blue mussel is characterized by two elongated and triangular shaped shell valves that are equal in size and have a bluish to black color. The shell valves are hinged together by means of a dorsal ligament (hinge) (Fig. 1b). The shell serves as skeleton attachment for the muscles (Fig. 1b). The part of the interior shell along which the mantle edges are attached, is called the pallial line. The mantle surrounds the pallial (mantle) cavity and is constituted of connective and gonadal tissue, the latter can be observed in Fig. 5. The pallial cavity is filled with seawater (pallial fluid). The pallial cavity is constituted of the infrabranchial (inhalant) and suprabranchial (exhalent) chamber, situated respectively ventrally and dorsally of the gill filaments and a dorsal recess, which is located dorsoposterior to the heart (partial reconstruction not shown). This dorsal recess is not distinguishable from the exterior of a live mussel since it is hidden underneath the hinge and dorsal mantle lining (Fig. 2b).

#### The posterior adductor muscle

**Figure 6:**
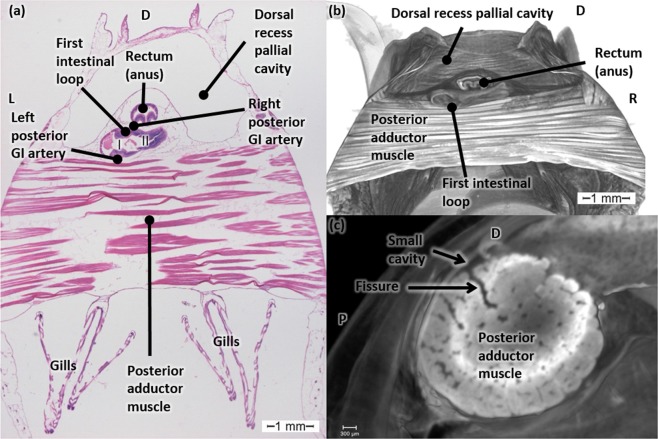
The posterior adductor muscle. (**a**) Transverse histological section of a Bouin fixated, H&E stained blue mussel (*Mytilus edulis*) at the level of the posterior adductor muscle. (**b**) Transverse micro-CT section in 3D at the level of the posterior adductor muscle of a critical point dried blue mussel after Bouin fixation. (**c**) Mediosagittal micro-CT section of the posterior adductor muscle of a PMA stained blue mussel. Abbreviations: GI, Gastro-intestinal; D, Dorsal; L, Left; R, Right; P, Posterior.

*Mytilus edulis* has a rudimentary anterior adductor muscle and a well-developed posterior adductor muscle (Fig. 6). The adductor muscles’ fibers run parallel from left to right.

As can be observed in Fig. 6a,b, the rectum ends in the anus dorsal to the posterior adductor muscle. In between the anus and the posterior adductor muscle, the first gastro-intestinal loop can be observed. On the mediosagittal section (Fig. 6c) of a PMA stained posterior adductor muscle, a clear fissure can be observed in all scanned mussels together with small spaces between the muscle fibers and some larger gaps. The fissure seems to be connected to a small cavity (Fig. 6c) at the dorsal outer border of the muscle. The spaces between the muscle fibers of the critical point dried mussels (Fig. 6a) are much larger compared to the posterior adductor muscles of PMA stained mussels (Fig. 6b,c).

#### The gastro-intestinal (GI) tract

**Figure 7:**
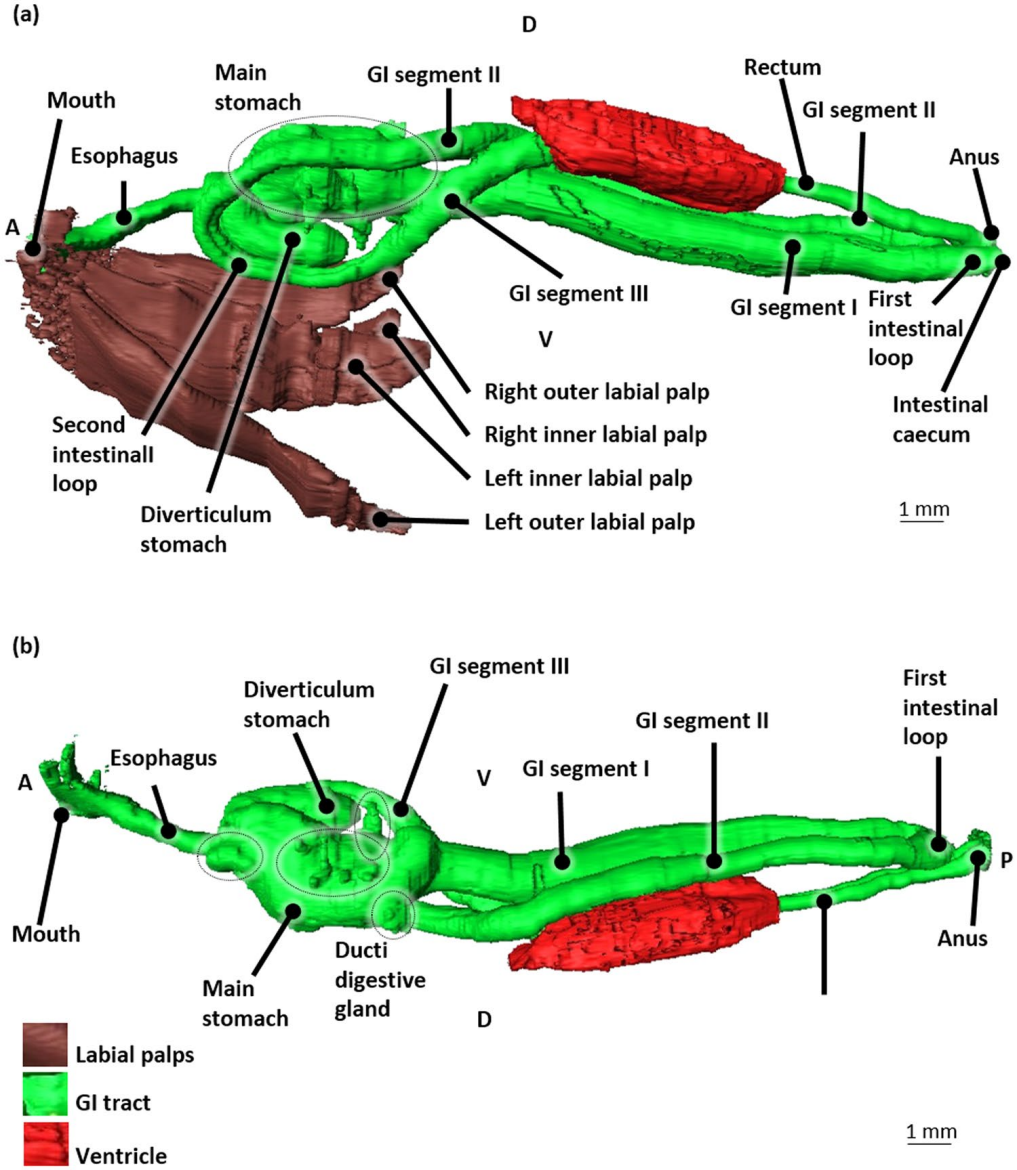
The gastro-intestinal (GI) tract and ventricle in 3D of a blue mussel (*Mytilus edulis*) based on histological sections of a Bouin fixated, H&E stained blue mussel. (**a**) Left lateral view, with labial palps. Dotted lines indicate the main stomach. (**b**) Right lateral view. Dotted lines indicate the ducts towards the digestive glad (the latter is not shown). Abbreviations: D, Dorsal; V, Ventral; A, Anterior; P, Posterior.

The mouth opening is bilaterally flanked by a pair of labial palps; the inner and outer labial palps (Fig. 7a). From the mouth, the GI tract continues in the esophagus, a straight tube leading towards the stomach (Fig. 7a,b).

The main stomach is cylindrical. To its anterior side it is connected to the esophagus, at its posterior side to the first GI segment and at its left anterior side to a large L-shaped blind ending protuberance which we will further nominate as diverticulum stomach (Fig. 7a). The main stomach is also connected with several ducts (ducti digestive gland) (Fig. 7b) to the digestive gland. The size of the latter occupies a great part of the dorsal body half.

From the main stomach, the intestinal tract continues posteriorly by a first GI segment (GI segment I) that has a blind caecum situated near the posterior adductor muscle close to the anus. The crystalline style could not be located. At the onset of the caecum, the first gastro-intestinal segment continues to the right by making a 180° turn creating a first intestinal loop. From this loop, the GI tract continues anteriorly as the second GI segment (GI segment II) and runs parallel to the GI segment I (Fig. 7a,b).

Posteriorly to the stomach, GI segment II crosses GI segment I dorsally and takes a turn to the left side of the body. It runs parallel to the left side of the main stomach until it turns ventrally, near the cranial part of the stomach to form the second intestinal loop.

It continues posteriorly at the left side of the diverticulum stomach as the third GI segment (GI segment III), then turns dorsally towards the ventricle of the heart. Once GI segment III penetrates the pericardial cavity and the ventricle of the heart (Fig. 7a,b), it is nominated as the rectum. The latter continues posteriorly parallel and dorsal to the GI segment I and II and finally ends in the anus (Fig. 6a,b) near the posterior adductor muscle.

#### The cardiovascular system

Supplementary Fig. S2a,b show the location of the heart (orange) in a micro-CT image. The heart is positioned in the middorsal line anterior to the posterior adductor muscle (Supplementary Fig. S2b).

The mussel heart consists of a single ventricle and two atria and is encased by the pericardium (Supplementary Fig. S3a–c). The ventricle has a single outlet to the anterior aorta and two inlets from the atria which contain the left and right atrioventricular valves, respectively. The atria surround the ventricle bilaterally and ventrally, as depicted in the dorsal and ventral views in Supplementary Fig. S3a,c. Ventro-lateral of the heart, the left and right atria are connected to the left and right oblique vein, respectively (Supplementary Fig. S3c).

The atrial wall presents multiple folds. The pericardial cavity is bilaterally connected to the pericardio-renal canals. The pericardio-renal canals run parallel to the oblique veins.

**Figure 8:**
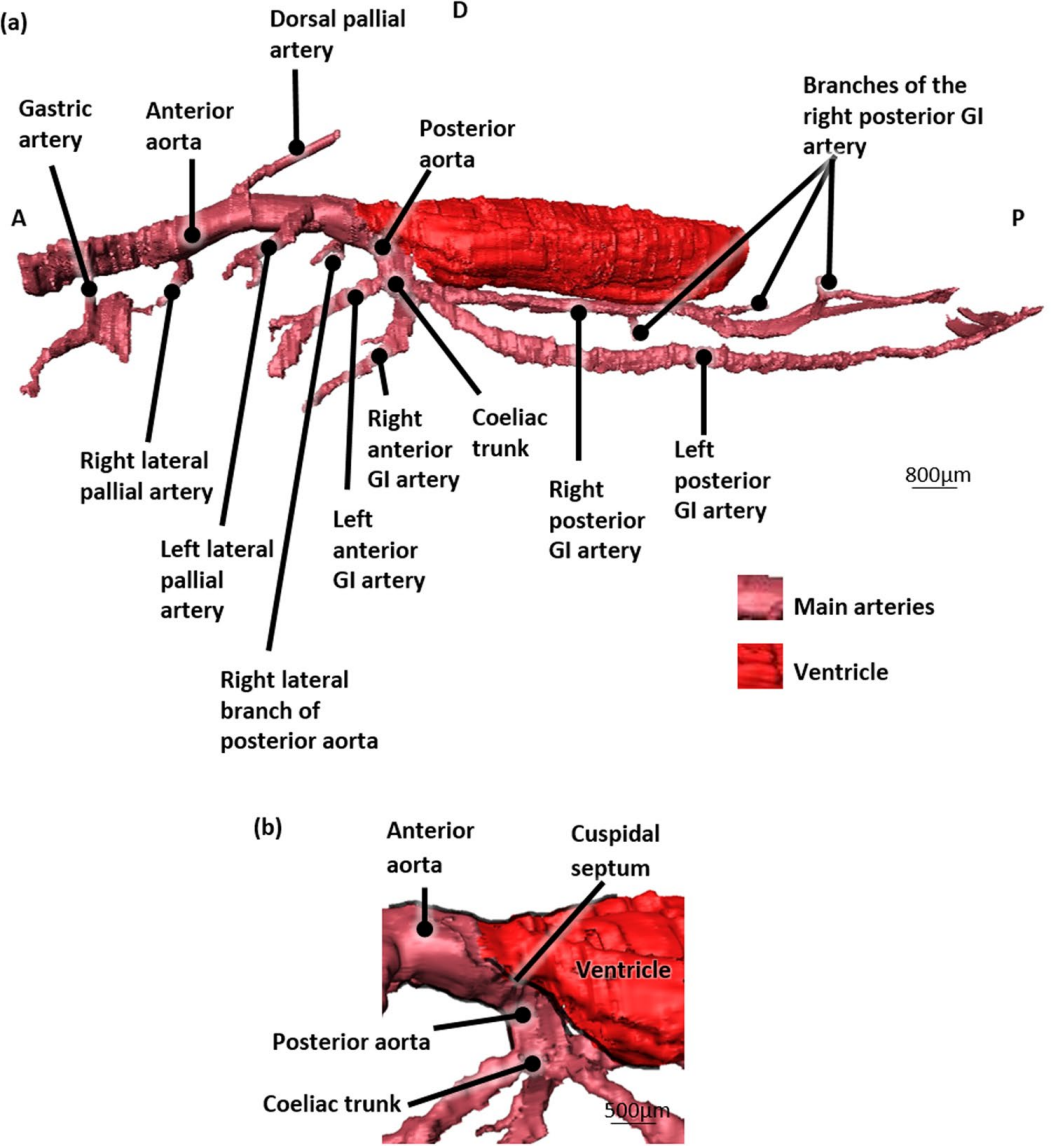
3D-visualization of the main arteries, based on histological section images of a Bouin fixated, H&E stained blue mussel (*Mytilus edulis*). (**a**) Left lateral view. (**b**) Left lateral view showing an enlargement of the ventricle outlet with the posterior aorta. Abbreviations: GI, Gastro-intestinal; D, Dorsal; A, Anterior; P, Posterior.

**Figure 9:**
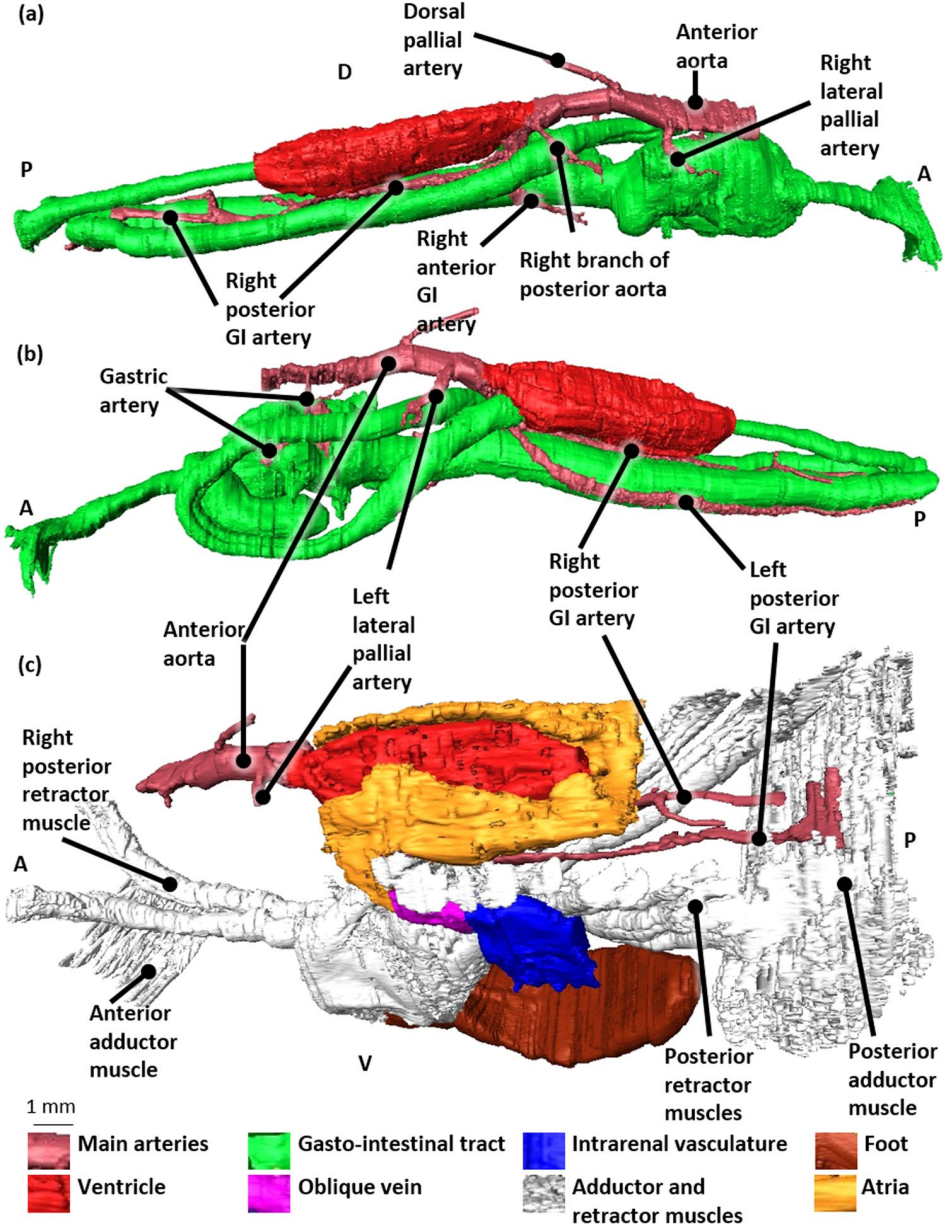
3D-visualization of the main arteries, based on histological section images of a Bouin fixated, H&E stained blue mussel (*Mytilus edulis*). (**a**) Right lateral view with the gastro-intestinal (GI) tract. (**b**) Left lateral view with the gastro-intestinal tract. (**c**) Left lateral view with the veins, muscles and foot. Abbreviations: GI, Gastro-intestinal; P, Posterior; A, Anterior; D, Dorsal; V, Ventral.

The anterior part of the ventricle gives rise to the anterior and posterior aorta (Fig. 8a). A cuspidal septum can be observed at the level of the anterior ventricle outlet (Fig. 8b). The posterior aorta leaves the ventricle at the same level as the anterior aorta and subsequently turns 180° to continue posteriorly adjacent and ventral to the ventricle (Fig. 8a,b). A small vessel branches from the right lateral side of this posterior aorta and extends towards the right mantle lobe (Figs. 8a and 9a). The posterior aorta continues further as the coeliac trunk and turns ventrally to give rise to the left and right anterior and to the left and right posterior GI arteries (Figs. 8a and 9a–c, and Supplementary Fig. S4).

The left anterior GI artery follows the first GI segment at its dorsal side and subsequently bifurcates into a left and right branch both adjacent to the first GI segment (Figs. 8a and 9a, and Supplementary Fig. S4).

The right anterior GI artery at first continues ventrally adjacent to the first GI segment and subsequently turns to the anterior side to branch into the intestinal gland (Figs. 8a and 9a, and Supplementary Fig. S4).

The posterior GI arteries both follow the first intestinal segment posteriorly (Figs. 8a, 9a and 9b).

The right posterior GI artery follows the right dorsolateral side of the first GI segment and has at least 3 side branches before it terminates in close proximity of the posterior adductor muscle (Figs. 8a and 9a–c).

The left posterior GI artery follows the first GI segment on its left lateral side and also ramifies several times along its way before it finally disperses into the posterior adductor muscle (Figs. 8a, 9b,c).

The anterior aorta continues to the anterior side of the mussel, dorsal to the stomach and branches along its way into (at least) three lateral branches and one dorsal branch (Figs. 8a, and 9a–c).

The first branch of the anterior aorta is the left lateral pallial artery. It leaves the anterior aorta halfway between the ventricle outlet and the dorsal pallial artery and can be traced into the left mantle lobe bifurcating in a lateral and medial branch (Figs. 8a, 9b,c).

The dorsal pallial artery immediately bends posteriorly after leaving the anterior aorta from its dorsal wall, towards the dorsal mantle lobe (Figs. 8a and 9a–c). The dorsal pallial artery subsequently bifurcates in a left and right dorsal pallial artery (Supplementary Fig. S4).

The third branch of the anterior aorta is the right lateral pallial artery and continues anteriorly (Figs. 8a, 9a, and Supplementary Fig. S4b).

At the level of the stomach a fourth branch leaves the anterior aorta; it is the onset of the gastric artery that bifurcates in a posterior and anterior gastric artery, both running adjacent to the left gastric wall (Figs. 8a and 9b).

The oblique veins run parallel to the pericardio-renal canals as described earlier (Supplementary Fig. S3b,c). The oblique vein is a perpetuation of part of the intrarenal vasculature (Supplementary Fig. S3b,c). The metanephridia (organ of Bojanus/excretory glands/kidneys) are constituted of the intrarenal vasculature and several cavities along the longitudinal axis of the mussel. As the excretory system of *Mytilus edulis* was not the scope of this research, the metanephridia were not visualized in 3D nor will be further described in this manuscript.

### Hemolymph withdrawal

Anesthesia with MgCl_2_ resulted in an opening of the shells for 0.5 cm, providing an easy access to the mussel’s adductor muscles.

Hemolymph could easily be extracted from the posterior adductor muscle (0.5–1.5 mL) (Supplementary Fig. S5) and more difficult from the less developed anterior adductor muscle (0.2 mL).

To reach the heart, a careful removal of the shell was preferred over the drilling of a hole in the shell as this latter procedure easily resulted in tissue damage. A 23G butterfly catheter could successfully be inserted peri-or intracardially (Supplementary Fig. S5 and S6) for extraction of a small (<0.2 mL) volume.

## Discussion

### 3D-visualization

Bouin fixative previously enabled a good preservation of the original anatomy in pig embryos^31^ and was also positively evaluated in this study both for preparation of histology as for micro-CT. However, for this study, artefacts were encountered e.g. in between the muscle fibers of the adductor muscles in the micro-CT images of the critical point dried specimens. Critical point drying is generally accepted as a non-destructive preparatory technique^32^. However, artefacts are reported due to mechanical forces during the preparation of a sample and during the dehydration process prior to critical point drying^33^. We believe that the dehydration process with ethanol and acetone might have induced the artefacts that were observed between the fibers of the adductor muscle.

PMA embedment after paraformaldehyde fixation in mouse (*Mus musculus*), zebrafish (*Danio rerio*) and clawed frog (*Xenopus laevis*) was described to result in qualitatively good visualization of and discrimination between tissue types and organs with micro-CT, including blood and blood vessels^34^. For this study however, the PMA embedment protocol was not sufficient to picture the mussel’s cardio-vascular anatomy. The PMA embedment period for the mussels was much shorter than as was described by Descamps *et al*.^34^ (6 h and 6 days, respectively). However, when we observe Supplementary Fig. S1, except for the circular lines in the posterior adductor muscle, there are no indications of an inadequate staining period. This observation suggests that a prolonged embedment in PMA would not further improve the visualization of the cardiovascular system. Bouin fixation in combination with critical point drying on the other hand resulted in a clear visualization of the heart and major arteries. This method was hence used to further study and describe the general mussel’s anatomy. However, the PMA staining of mussels was preferred to study the posterior adductor muscle, hereby avoiding false conclusions based on the artefacts mentioned earlier.

### The anatomy of the blue mussel *Mytilus edulis*

The existence of an infrabranchial and suprabranchial chamber^35^, inhalant syphon and exhalent syphon^30^, all part of the pallial cavity, was confirmed by our findings. However, before this study, no description was available of the dorsal recess of this pallial cavity. Its anatomical position poses a risk to contaminate hemolymph extracted from the pericardial cavity or ventricle with pallial fluid.

Pallial fluid (seawater) should not be confused with extrapallial fluid which has a composition very similar to hemolymph. Extrapallial fluid can be found in the tiny space (extrapallial space) enclosed between the shell and mantle and is involved in shell formation^36^. Its extraction is described in different species including *Mytilus edulis*^9,37^. As both micro-CT and histological procedures required the removal of both shells, it was not possible to visualize the extrapallial space in 3D for this study.

The posterior adductor muscle is the most commonly used hemolymph extraction site, due to its accessibility (e.g. with a needle through the exhalent syphon). It is however not clear where the hemolymph comes from. Following the description of Sabatier^38^ which was confirmed by Purdie^29^ (a ‘*sinus among the fibers of the posterior adductor muscle’*), the hemolymph that is withdrawn from the posterior adductor muscle could be punctured from hemolymph perfusions in between the muscle fibers. Although the excessive lacunar spaces between the muscle fibers of the critical point dried mussels are presumptive artefacts caused by the dehydration process as discussed above, the small spaces, gaps and fissures between the muscle fibers on the micro-CT images of the PMA-stained mussels are apparently physiological. Moreover, the small cavity at the dorsal border of the posterior adductor muscle connected to the large fissure in the adductor muscle indicates a connection to an afferent or efferent vascular vessel, presumably branch(es) of a GI artery. It is our hypothesis that the hemolymph withdrawn from the posterior adductor muscle is withdrawn from blood in the final branches of the left (and right) posterior GI arteries. Which lines with Field^39^, describing a pair of large vessels arising from the ventrolateral surfaces of the aortic bulb and subdividing into numerous branches supplying amongst others the posterior adductor muscle. Especially the left posterior GI branch can be discerned. This branch is remarkably wide and flattened upon its close contact with the posterior adductor muscle. The latter indicates that it could be readily dispersed into the posterior adductor muscle, providing it from hemolymph which could explain the ease of hemolymph withdrawal upon puncturing. Based on the 3D-reconstructions, the location of the small cavity reconciles with the position of the termination of the left posterior GI artery, favoring our hypothesis. However, this could not be confirmed based on histological sections probably due to the preparation process.

The description of Sabatier^38^ of the intestinal tract diverges slightly from what was observed in this study. Merely some denominations were revised, since they were neither mentioned nor described as such by Gosling^35^ or Bayne^30^. In short, the dilatated utricular stomach according to Sabatier^38^ was denominated as main stomach in this study and the tubular stomach^38^ or direct intestine according to Field^39^, was renamed as intestinal segment I. The large L-shaped blind-ending protrusion of the main stomach was termed diverticulum stomach in this study. It was not described before, although Sabatier^38^ did mention a stomachal diverticulum of 5–6 mm extending from the lower part of the stomach that was according to Field^39^ not regularly present. The crystalline style could be traced neither on histological sections nor with micro-CT. Since the crystalline style releases enzymes for digestion^40^, it can disappear within a couple of days in laboratory conditions^38^. The short caecum at the end of GI segment I, near the posterior adductor muscle was described by Sabatier^38^ and Field^39^ and its presence was confirmed by our findings. From the intestinal segment II onwards till the anus, including the penetration of the ventricle by the rectum, the morphological findings in this study reconcile with the extensive and detailed descriptions of Sabatier^38^.

The heart of *Mytilus edulis* has a very thin vascular lining in contrast to a thick-walled muscular heart of vertebrates. The innumerable involutions and folds of the atrial wall were reported before and are suspected to ultra-filtrate atrial hemolymph as a pericardial gland during atrial pressure build-up^41,42^. The presence of the pericardio-renal canal, connecting the pericard to each metanephridium, favors this hypothesis. The position of the atrio-ventricular valves and posterior connection between the left and right atria^39,42^ could be confirmed with histological sections and 3D-visualization.

There is some contradiction regarding the location and existence of an aortic bulb (enlargement of the aorta at its point of origin from the heart) in *Mytilus edulis*. According to Purdie^29^, the aorta leaves the ventricle at the anterior end and soon widens out into a large aortic bulb which gives rise to several arteries. Fox^43^, in contrast, described the aortic bulb as a swelling of the anterior part of the ventricle with the rectum passing through the center of this bulb and the aorta leaving the bulb on its dorsal anterior wall. According to Field^39^ the aortic bulb arises immediately below and just anterior to the point where the rectum penetrates into the ventricle. Based on our findings we could not confirm the existence of an aortic bulb, neither as described by Purdie^16^,Fox^43^ nor Field^39^. Moreover, the transition from ventricle to aorta occurs according to the 3D reconstruction dorsal to the point where the rectum penetrates the ventricle and not below as described by Field^39^. In addition, in our observations, we saw the presence of a cuspidal septum at the level of the anterior ventricle outlet, which has never been described before. This septum could be a valve with at least one cusp, serving to prevent reflux from the aortae to the ventricle during ventricular diastole. Indeed, in literature, the existence of ‘different kind of devices’ present at the junction of the ventricle with the atria and the aorta is reported^44^. The latter author might refer to, besides the atrio-ventricular valves, the presence of a ventriculo-aortic valve, which would coincide with our findings.

A posterior aorta is described in many bivalve mussel species. It was described in *Mytilus edulis* as a very small insignificant artery supplying only the floor of the pericardium^29^, which stands in sharp contrast to the large, though short blood vessel that is observed in this study. The posterior aorta continues as the coeliac trunk from the split-off of the right branch of the posterior aorta and its division into the four GI arteries. The latter were described by Purdie^29^ and Field^39^ as two GI arteries that subsequently divide into the anterior and posterior GI arteries.

The venous system of bivalves is very complex and varies from unwalled lacunae and hemal spaces to definite lumina surrounded by manifest venous walls^45^. This might explain why only the oblique veins could be differentiated with certitude in this study. According to Purdie^29^, great parts of the venous system of mussels consist of lacunae and hemal spaces. Indeed, as was observed with histologic sections in this study, dispersed parts of the visceral mass and mantle are constituted of lacunar tissue. It was not possible to clearly identify the intrarenal vasculature of *Mytilus edulis*, however since a connection was found between the oblique vein and the metanephridium, at least part of the intrarenal vasculature has to be allocated as venous. Indeed literature states that nearly all hemolymph passes the metanephridia before it finds its way via the oblique veins to the atria of the heart^29^. Sabatier^38^ describes a longitudinal vein, frequently being interrupted by masses of the excretory tissue, suggesting that part of the intrarenal vasculature observed in this study might be allocated as the longitudinal vein.

### Hemolymph withdrawal

Anesthesia for adductor muscle relaxation (e.g. MgCl_2_, MS222) has been described in several bivalves such as oysters and scallops with varying degrees of success^46,47^. MgCl_2_ was successfully applied in this study as mussel anesthetic and allowed a clear visualization and manipulation of the mussel’s adductor muscles without the necessity to use of a knife or plier to prise the valves apart or to create a notch in the shells as described^48^. Hemolymph withdrawal from the anterior adductor muscle was not preferred due to the limited extracted hemolymph volume. This is in sharp contrast to the hemolymph extraction results from this same site in the fresh water mussel *Elliptio complanata* whose anterior adductor muscle is well developed^27^.

Withdrawal from the heart was more difficult compared to extraction from the adductor muscles because of the dorsal location of the heart close to the hinge. Taken into account the new insights from this study, it should be possible to puncture the pericardial cavity and the different heart structures without drilling a hole or removing a shell, after sedation of the mussels. This could be done by inserting a needle (23 to 26G) right posteriorly of the hinge, into the pericardial cavity or into the ventricle. A similar non-invasive method was reported for non-sedated brackish water clams (*Rangia cuneata*) by blind injection through the hinge into the pericardial cavity^8^. The latter however, creates a considerable contamination risk of the sample with ventricular fluid^27^ and/or pallial fluid from the dorsal recess of the pallial cavity if the animal is not properly drained (own findings, results not shown). This blind method of puncturing through the hinge as described supra could result in puncturing the anterior aorta rather than the heart in case of *Mytilus edulis*.

Due to the brittle nature of the cardiac tissue, drilling a hole in the shell was not possible without injuring the mussel as Friedl *et al*.^11^ successfully did with the American oyster *Crassostrea virginica*. However, one shell half could be successfully removed, allowing us to puncture both the pericardial cavity and the ventricle, under a stereomicroscope. The latter method was reported before by Gustafson *et al*.^27^ and Fyhn & Costlow^8^ in the freshwater mussel (*Elliptio complanata*) and the brackish water clam (*Rangia cuneata*), respectively.

In conclusion, in this study we visualized the anatomy of the blue mussel for the first time (in 3D) with state of the art techniques. We further described the anatomy and made comparison to the pioneering work of Sabatier^38^ and Purdie^29^ on *Mytilus* sp. The anatomical descriptions and 3D visualizations developed for this study will be of great value in many research fields that focus on mussels and may be useful for a better understanding of other bivalves too. Finally we focused on the hemolymph system. Through the obtained 3D-visualization based on micro-CT and histological sections, the different hemolymph extraction sites were clearly mapped. These data provide an insight from where hemolymph withdrawn from the posterior adductor muscle could be punctured from. Our data suggest that this hemolymph would be withdrawn from perfusions from small spaces and fissures between the muscle fibers that are connected to at least one supplying artery, the left posterior gastro-intestinal artery. Based on our findings, non-invasive hemolymph extraction should be possible from the heart (ventricle and pericardial cavity) in a sedated mussel (MgCl_2_). However, caution should be taken for contamination with intestinal content and microflora from the first intestinal loop and rectum. In addition, contamination with seawater from the dorsal recess of the pallial cavity can be avoided by drainage of the pallial fluid prior to hemolymph extraction is therefore essential. Finally, hemolymph extraction from the anterior aorta is less straightforward due to its location behind the hinge. These data describe and evaluate different hemolymph extraction locations and techniques that could be applied in a wide range of research activities and meanwhile demonstrate the wide aspect of the 3D images developed of the blue mussel for this study.

## Materials and methods

### Mussels

Sixty-five healthy blue mussels (*M. edulis* Linnaeus, sizes 3 ± 1 cm) were collected from Yerzeke (The Netherlands), and from Ostend (Belgium). These mussels were included for the 3D-reconstruction applying histology and µCT-imaging, as well as for injection protocols, hemolymph analyses of puncture sites, and separate histological sections for verification of the findings as described further.

### 3D-visualization based on histological section images

Ten mussels were euthanized by immersion for 3 h in seawater supplemented with 0.2% (v/v) benzocaine (10% (m/v) ethylaminobenzoate in acetone (Sigma-Aldrich)). Two fixation methods were applied. The mussels were placed either in a phosphate-buffered 4% formaldehyde solution at room temperature for 48 h and transferred to pure water for 8 h, or in Bouin’s solution (150 mL saturated aqueous picric acid (30 g L^−1^), 50 mL formaldehyde 35%, 10 mL acetic acid (glacial) overnight) and transferred to 70% ethanol for 48 h. As removing mussels from the shell prior to fixation results in small to considerable muscle damage (results not shown), the mussels remained in the shells during fixation. After fixation, the mussels were carefully removed from their protective shells and subsequently rinsed several times in 70% ethanol baths to rinse out the yellow color of the Bouin’s solution. Afterwards, they were placed in an automated system (Shandon Citadel 1000 histokinette, 20 h cycle duration) in which they were dehydrated in a series of alcohols (70% ethanol, 80% ethanol, 94% ethanol, and isopropyl alcohol), cleared in xylene and impregnated with paraffin under vacuum. Finally, the mussels were embedded in paraffin wax (Microm EC 350-1 embedding station, Prosan, Merelbeke, Belgium) to allow transverse serial sections (10 µm) using a microtome (Microm microtome HM 360, Prosan). Sections were stained with hematoxylin and eosin (H&E) and further processed according to standard laboratory protocols.

All sections of the mussels selected for reconstruction were evaluated using a light microscope (Olympus BX61). Record was kept of all slices that were not suitable for 3D-reconstruction due to technical artefacts such as folds and cracks. All sections were digitalized (Olympus DP50). As the tissue samples were large, several pictures had to be taken in order to cover each section in its entirety. By means of the multiple image alignment module (MIA) of analysis software (Cell F, Olympus Soft Imaging Solutions), these individual images were automatically merged into one single picture. A white scale of invariable dimension was added to every digital image to ensure a constant width and height of every picture as required by the reconstructive software. This resulted in 502 pictures with a pixel size of 10.05 ×10.05.

Amira™ (FEI, RRID:SCR_007353), an image segmentation and 3D-surface generating software package, was used for segmentation of the obtained reconstructed pictures. Manual segmentation was done according to Cornillie, Van den Broeck, & Simoens^31^.

### Micro-CT

#### Preparation methods

A first preparation method used Bouin fixation followed by a critical point drying process. Ten mussels were euthanized and fixated overnight in Bouin’s solution. The shells were carefully removed and subsequently the mussels were dehydrated (as described before^48^) in an increasing alcohol series followed by increasing ethanol–acetone series up to 100% acetone. The samples were then dried to the critical point with a Balzers CPD 030 critical point drier (Sercolab bvba, Merksem, Belgium) for further scanning with micro-CT.

In a second preparation method, phosphomolybdic acid (PMA) staining was used as X-ray contrast enhancer. Ten mussels were placed in a sea water bath for 30 minutes to allow filtration of the water. At the posterior end, a blunt object (3 mm diameter) was placed between the valves to prevent the shells from closing. Ten other mussels were anesthetized with MgCl_2_ (28 g L^−1^, maintaining a salinity of 35 g L^−1^). Subsequently, all mussels were stained and fixated as described by Descamps *et al*.^34^ with some changes. In short, all mussels were placed in PMA (2.5% solution in demineralized water) for 6 h, allowing PMA to penetrate the mussel tissues, followed by fixation in 4% paraformaldehyde until scanning with micro-CT took place (6 days).

The above described specimens were subsequently scanned at the micro-CT-scanning facilities of UGCT (Ghent University, Belgium). The custom-built X-ray micro-CT-scanner of medium energy (up to 160 kV) achieved feature recognition of 2 μm on small samples, as specified by the X-ray tube manufacturer^49^. The tube was operated at 70 kV at 25 W, using a one millimeter aluminium filtration to optimize the signal-to-noise ratio for this sample size and composition. 1800 shadow images (2000 ×2000 pixels) were recorded covering 360° within about 30 minutes measurement time. The back-projection calculations to obtain the reconstructed images were made with the Octopus custom-made software package. The voxel pitch of the isotropically sampled 3D-dataset was about 25 µm.

#### Hemolymph withdrawal

All mussels used for hemolymph withdrawal were anesthetized with MgCl_2_ (28 g L^−1^, maintaining a salinity of 35 g L^−1^), until the valves did not close upon contact, after approximately 30 minutes. Contamination with pallial fluid (seawater) was minimized by carefully draining the mussels prior to hemolymph extraction by holding the mussel facing up its posterior side in dorsal view, with the mussel’s anterior side resting on a bed of paper in the hand palm.

Hemolymph extraction from the posterior and anterior adductor muscle of ten mussels was performed using a 23G needle, with a 1 mL syringe. The posterior adductor muscle was punctured through the exhalent syphon, in the center of the muscular bundle as can be observed in Supplementary Fig. S5.

Withdrawing hemolymph directly from the heart was done in two ways.

In a first method, a hole was drilled unilaterally in the shell of five mussels, dorsal to the location of the heart, using a 2 mm cordless drill (Powxq5243 Powerplus XQ). Subsequently, a 23G butterfly catheter was inserted in the heart.

In a second method, one of the shell valves was removed. To do so, the posterior adductor muscle of 10 mussels was cut at its attachment to one shell half. The mantle was carefully removed from the right shell, followed by the removal of this shell. The dorsal midline, where the beating heart is located, was visualized using a stereomicroscope (Olympus SZX7 with an Olympus Soft Imaging System colour View I Camera). Different sizes of needles, ranging from 21 to 26G, were tested, including a butterfly catheter of 23G attached to a 1 mL syringe (Supplementary Fig. S6). The 21G needle was too thick as it destroyed the fragile heart tissue upon insertion, while applying a 26G needle resulted in easy access to the heart tissue.

## Supplementary information

Supplementary Movie.

Supplementary Figures.

## References

[1] Amachree D, Moody AJ, Handy RD (2013). Comparison of intermittent and continuous exposures to cadmium in the blue mussel, *Mytilus edulis*: accumulation and sub-lethal physiological effects. Ecotox. Environ. Safe..

[2] Browne MA, Dissanayake A, Galloway TS, Lowe DM, Thompson RC (2008). Ingested microscopic plastic translocates to the circulatory system of the mussel, *Mytilus edulis* (L.). Environ. Sci. Technol..

[3] Ivanina AV, Hawkins C, Sokolova IM (2016). Interactive effects of copper exposure and environmental hypercapnia on immune functions of marine bivalves *Crassostrea virginica* and *Mercenaria mercenaria*. Fish Shellfish Immun..

[4] Han ZX, Wu DD, Wu J, Lv CX, Liu YR (2014). Effects of ocean acidification on toxicity of heavy metals in the bivalve *Mytilus edulis* L. Synth React. Inorg. M..

[5] Pipe RK, Coles JA, Carissan FMM, Ramanathan K (1999). Copper induced immunomodulation in the marine mussel, *Mytilus edulis*. Aquat. Toxicol..

[6] Sheir SK, Handy RD, Henry TB (2013). Effect of pollution history on immunological responses and organ histology in the marine mussel *Mytilus edulis* exposed to cadmium. Arch. Environ. Con. Tox..

[7] Van Cauwenberghe L, Janssen CR (2014). Microplastics in bivalves cultured for human consumption. Environ. Pollut..

[8] Fyhn HJ, Costlow JD (1975). Anaerobic sampling of body-fluids in bivalve mollusks. Comp. Biochem. Phys. A.

[9] Zittier ZMC, Bock C, Lannig G, Portner HO (2015). Impact of ocean acidification on thermal tolerance and acid-base regulation of *Mytilus edulis* (L.) from the North Sea. J. Exp. Mar. Biol. Ecol..

[10] Martin AW, Harrison FM, Huston MJ, Stewart DM (1958). The blood volumes of some representative molluscs. J. Exp. Biol..

[11] Friedl FE, Alvarez MR, Johnson JS, Gratzner HG (1988). Cytometric investigations on hemocytes of the American oyster, *Crassostrea virginica*. Tissue Cell.

[12] Picken LER (1937). The mechanism of urine formation in invertebrates. II.The excretory mechanism in certain mollusca. J. Exp. Biol..

[13] Cochennec-Laureau N, Auffret M, Renault T, Langlade A (2003). Changes in circulating and tissue-infiltrating hemocyte parameters of European flat oysters, *Ostrea edulis*, naturally infected with *Bonamia ostreae*. J. Invertebr. Pathol..

[14] Lemaitre B, Hoffmann J (2007). The host defense of *Drosophila melanogaster*. Annu. Rev. Immunol..

[15] Ottaviani E (2011). Immunocyte: the invertebrate counterpart of the vertebrate macrophage. Inv Surv. J..

[16] Song, L., Wang, L., Qiu, L., & Zhang, H. Bivalve Immunity in *Invertebrate Immunity. Advances in Experimental Medicine and Biology vol 708* (eds. Söderhäll, K.) 44–65 (Springer, Boston, MA, 2010). 10.1007/978-1-4419-8059-5_3 21528692

[17] Pallavicini A (2008). High sequence variability of myticin transcripts in hemocytes of immune-stimulated mussels suggests ancient host-pathogen interactions. Dev. Comp. Immunol..

[18] Martinez-Lopez, A. *et al*. pH-dependent solution structure and activity of a reduced form of the host-defense peptide myticin C (Myt C) from the mussel *Mytilus galloprovincialis*. *Mar Drugs* **11**(7), 2328–2346, Jul 4, 10.3390/md11072328 (2013). 10.3390/md11072328 PMC3736426 23880927

[19] Balseiro P (2011). *Mytilus galloprovincialis* myticin C: a chemotactic molecule with antiviral activity and immunoregulatory properties. Plos One.

[20] Novoa B (2016). Antiviral activity of myticin C peptide from mussel: an ancient defence against herpesviruses. J. Virol..

[21] Jemaà M (2014). Adult somatic progenitor cells and hematopoiesis in oysters. J. Exp. Biol..

[22] Li Y (2017). The hematopoiesis in gill and its role in the immune response of Pacific oyster *Crassostrea gigas* against secondary challenge with *Vibrio splendidus*. Dev. Comp. Immunol..

[23] Galloway JL, Zon LI (2003). Ontogeny of hematopoiesis: examining the emergence of hematopoietic cells in the vertebrate embryo. Curr. Top. Dev. Biol..

[24] Lin X, Söderhäll I (2011). Crustacean hematopoiesis and the astakine cytokines. Blood.

[25] Orkin SH, Zon LI (2008). Hematopoiesis: an evolving paradigm for stem cell biology. Cell..

[26] Cummings, K. S. & Graf, D. L. Mollusca: bivalves in *Ecology and Classification of North American* Freshwater *Invertebrates 3rd* (eds. Thorp, J. & Covich, A.) 309–384 (Academic Press, 2009).

[27] Gustafson LL (2005). Evaluation of a nonlethal technique for hemolymph collection in *Elliptio complanata*, a freshwater bivalve (Mollusca: Unionidae). Dis. Aquat. Organ..

[28] Norton, J. H. & Jones, G. W. In *The Giant Clam: an Anatomical and Histological Atlas* 1–144 (ACIAR Monographs, 1992).

[29] Purdie, A. In *The Anatomy of the Common Mussels: (Mytilus Latus, Edulis, and Magellanicus*) (ed. Purdie, A.) 1–45 (Wentworth Press, 1887).

[30] Bayne, B. L. In *Marine Mussels: Their Ecology and* Physiology 1st *edition* (ed. Bayne, B. L.) 1–506 (Cambridge University Press, 1976).

[31] Cornillie P, Van den Broeck W, Simoens P (2008). Three-dimensional reconstruction of the remodeling of the systemic vasculature in early pig embryos. Microscop. Res. Techniq..

[32] Fischer, E. R., Hansen, B. T., Nair, V., Hoyt, F. H. & Dorward, D. W. Scanning Electron Microscopy. *Curr. Protoc. Microbiol*. **Chapter 2**, Unit 2B.2 (2012). 10.1002/9780471729259.mc02b02s25 PMC3352184 22549162

[33] Bahr GF, Engler WF (1980). Artifacts observed in critical-point-dried preparations of human chromosomes by electron microscopy. J. Ultrastruct. Res..

[34] Descamps E (2014). Soft tissue discrimination with contrast agents using micro-CT scanning. Belg. J. Zool..

[35] Gosling, E. M. Morphology of bivalves in *Bivalve Molluscs: Biology, Ecology, and Culture* (ed. Gosling, E. M.) 7–43 (Blackwell Publishing Ltd, 2003).

[36] Crenshaw MA (1972). The inorganic composition of molluscan extrapallial fluid. Biol. Bull..

[37] Thomsen J (2010). Calcifying invertebrates succeed in a naturally CO_2_-rich coastal habitat but are threatened by high levels of future acidification. Biogeosciences.

[38] Sabatier, A. In *Etudes sur la Moule Commune (Mytilus edulis)* (eds. Coulet, C. & Delahaye, V.-A.) 1–150 (Montpellier, 1877).

[39] Field, I. A. Biology and economic value of the sea mussel Mytilus edulis. *Bull US Bur Fish* **38**, 127–259 (1921–1922).

[40] Gros O, Frenkiel L, Felbeck H (2000). Sulfur-oxidizing endosymbiosis in *Divaricella quadrisulcata* (Bivalvia: Lucinidae): morphological, ultrastructural, and phylogenetic analysis. Symbiosis.

[41] Andrews EB, Jennings KH (1993). The anatomical and ultrastructural basis of primary urine formation in bivalve mollusks. J. Mollus. Stud..

[42] Seo E (2014). Magnetic resonance imaging analysis of water flow in the mantle cavity of live *Mytilus galloprovincialis*. J. Exp. Biol..

[43] Fox, R. *Invertebrate anatomy online: Mytilus edulis*, http://lanwebs.lander.edu/faculty/rsfox/invertebrates/mytilus.html (2006).

[44] Narain AS (1976). A review of the structure of the heart of molluscs, particularly bivalves, in relation to cardiac function. J. Mollus. Stud..

[45] Milne-Edwards, M. Observations et expériences sur la circulation chez les mollusques in *Annales des Sciences Naturelles, Zoologie et Biologie Animale, IIIe Série* (ed. Fortin Massón, C.) 289–320 (Imprimerie de Bourgogne et Martinet, 1845).

[46] Lewbart GA, Mosley C (2012). Clinical anesthesia and analgesia in invertebrates. J. Exot. Pet Med..

[47] Ross, L. G. & Ross, B. Anaesthesia of aquatic invertebrates in *Anaesthetic and Sedative Techniques for Aquatic Animals. 3rd ed*. (eds. Ross, L. G. & Ross, B.) 176–178 (Blackwell Publishing Ltd., 2008).

[48] De Spiegelaere W, Casteleyn C, Van den Broeck W, Simoens P (2008). Electron microscopic study of the porcine choroid plexus epithelium. Anat. Histol. Embryol..

[49] Masschaele B (2013). HECTOR: a 240 kV micro-CT setup optimized for research. J. Phys. Conf. Ser..

[50] Ford SE (1986). Effect of repeated hemolymph sampling on growth, mortality, hemolymph protein and parasitism of oysters, *Crassostrea virginica*. Comp. Biochem. Phys. A.

[51] Yanick JF, Heath DD (2000). Survival and growth of mussels subsequent to hemolymph sampling for DNA. J. Shellfish Res..

[52] Moreira FT, Browne MA, Coleman RA (2013). Effect of extraction-method, period of incubation and tidal emersion on the viability of haemocytes from oysters. Mar. Pollut. Bull..

[53] Al-Subiai SN, Jha AN, Moody AJ (2009). Contamination of bivalve haemolymph samples by adductor muscle components: implications for biomarker studies. Ecotoxicology.

[54] Calvo-Iglesias J (2016). Characterization of a monoclonal antibody directed against *Mytilus* spp larvae reveals an antigen involved in shell biomineralization. Plos One.

