# Supplementary material for: The blue mussel inside: 3D visualization and description of the vascular-related anatomy of Mytilus edulis to unravel hemolymph extraction
Source: Sci Rep. 2020 Apr 21;10:6773. doi: 10.1038/s41598-020-62933-9 (PMC7174403; doi:10.1038/s41598-020-62933-9)
Supplement: Supplementary file 2 — Supplementary Figures. [file 41598_2020_62933_MOESM2_ESM.pdf]

Supplementary Figures legends and figures

Supplementary Figure S1 : Mediosagittal micro-CT section in 3D at the level of the heart of a PMA stained mussel (*Mytilus edulis*). Abbreviations: GI, Gastro-intestinal; P, Posterior; A, Anterior; D: Dorsal; V, Ventral.

Supplementary Figure S2 : Location of the heart (orange) of the blue mussel (*Mytilus edulis*) in a micro-CT image of a critical point dried mussel after Bouin fixation. (a) Right lateral view (b) Dorsal view. Abbreviations: L, Left; R, Right; P, Posterior; A, Anterior; D, Dorsal; V, Ventral.

Supplementary Figure S3 : 3D-visualization of heart structures, based on histological section images of a Bouin fixated, H&E stained blue mussel (*Mytilus edulis*). (a) and (b) Dorsal view. (c) Ventral view. Abbreviations: L, Left; R, Right; P, Posterior; A, Anterior.

Supplementary Figure S4 : Transverse histological section of a Bouin fixated, H&E stained mussel (*Mytilus edulis*) at the level of the cuspidal septum of the heart. Abbreviations: I, first gastro-intestinal segment; II, second gastro-intestinal segment; III, third gastro-intestinal segment; GI, Gastro-intestinal; L, Left; R, Right; D, Dorsal.

Supplementary Figure S5 : Hemolymph withdrawal from the posterior adductor muscle in the blue mussel (*Mytilus edulis)*. Abbreviations: D, Dorsal; V, Ventral.

Supplementary Figure S6 : Hemolymph withdrawal from the heart in the blue mussel (*Mytilus edulis)*.

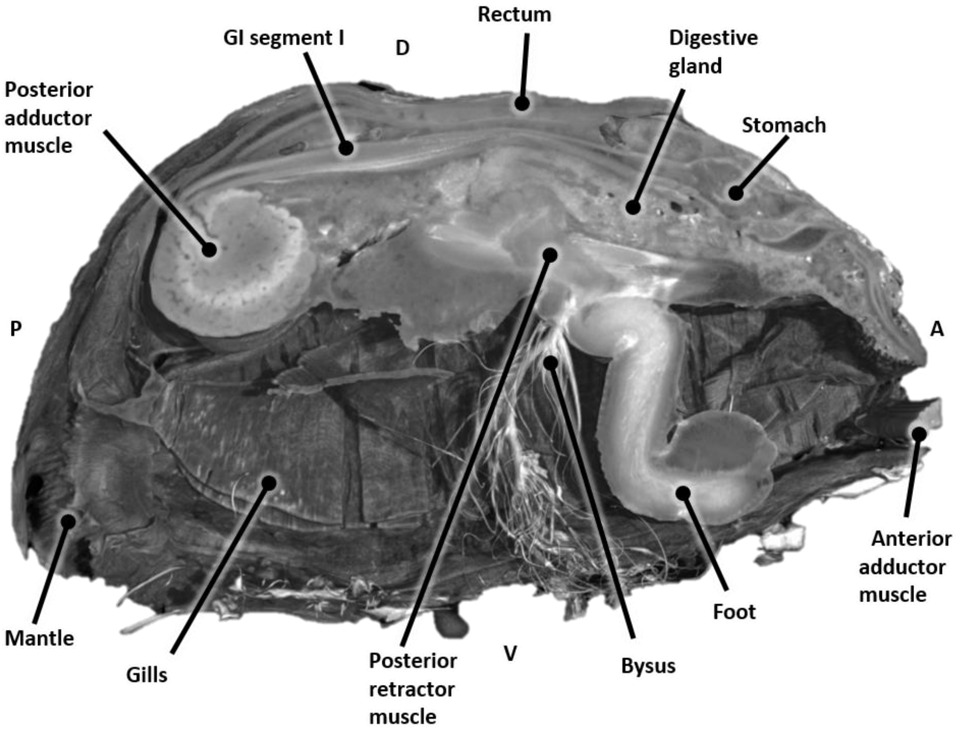

Supplementary Figure S1 : Mediosagittal micro-CT section in 3D at the level of the heart of a PMA stained mussel (*Mytilus edulis*). Abbreviations: GI, Gastro-intestinal; P, Posterior; A, Anterior; D: Dorsal; V, Ventral.

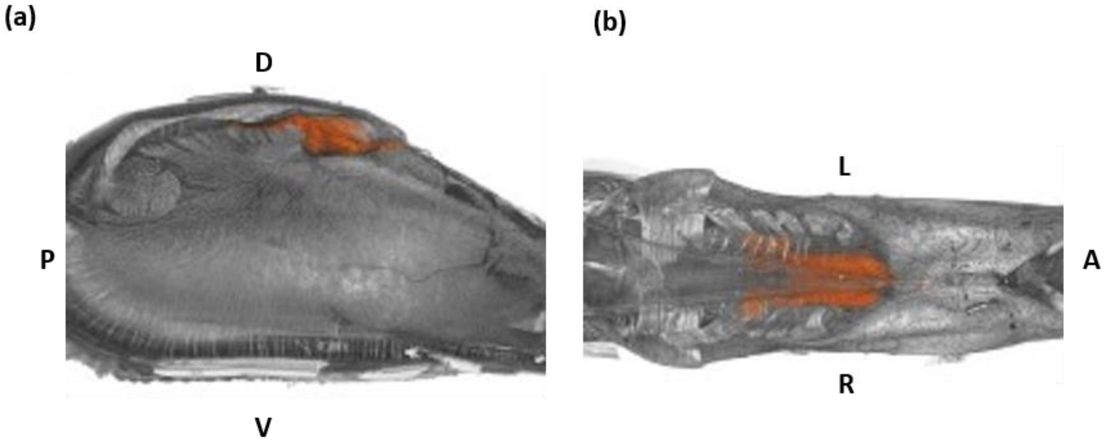

Supplementary Figure S2 : Location of the heart (orange) of the blue mussel (*Mytilus edulis*) in a micro-CT image of a critical point dried mussel after Bouin fixation. (a) Right lateral view (b) Dorsal view. Abbreviations: L, Left; R, Right; P, Posterior; A, Anterior; D, Dorsal; V, Ventral.

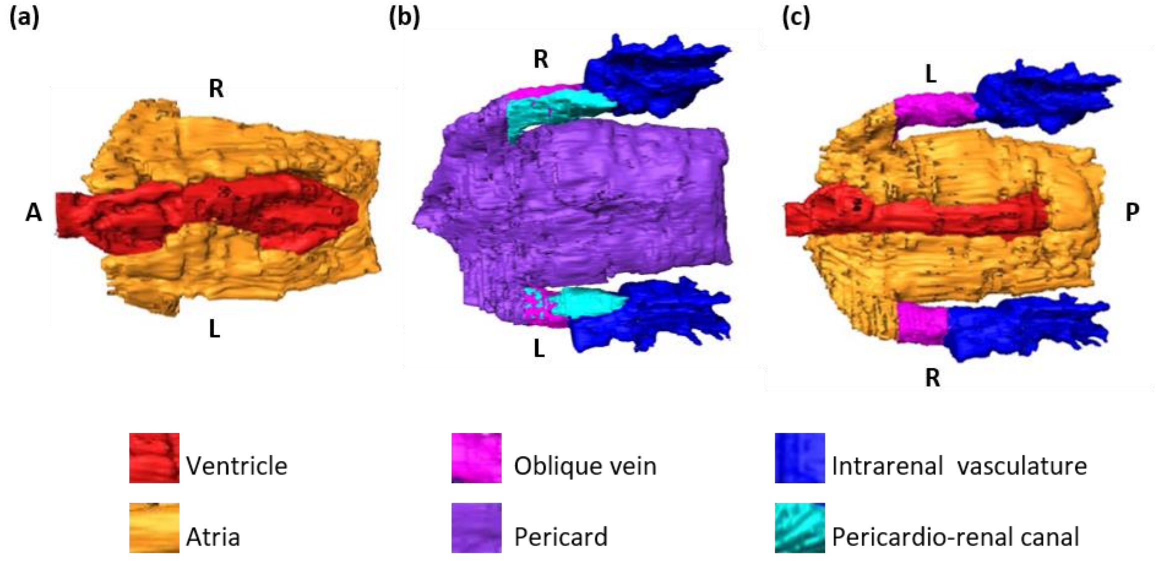

Supplementary Figure S3 : 3D-visualization of heart structures, based on histological section images of a Bouin fixated, H&E stained blue mussel (*Mytilus edulis*). (a) and (b) Dorsal view. (c) Ventral view. Abbreviations: L, Left; R, Right; P, Posterior; A, Anterior.

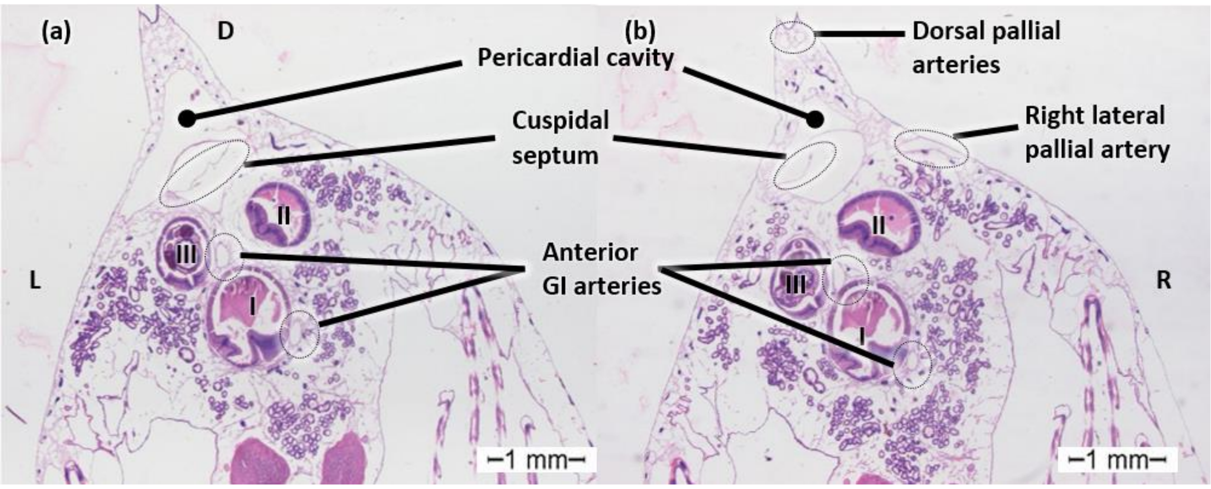

Supplementary Figure S4 : Transverse histological section of a Bouin fixated, H&E stained mussel (*Mytilus edulis*) at the level of the cuspidal septum of the heart. Abbreviations: I, first gastro-intestinal segment; II, second gastro-intestinal segment; III, third gastro-intestinal segment; GI, Gastro-intestinal; L, Left; R, Right; D, Dorsal.

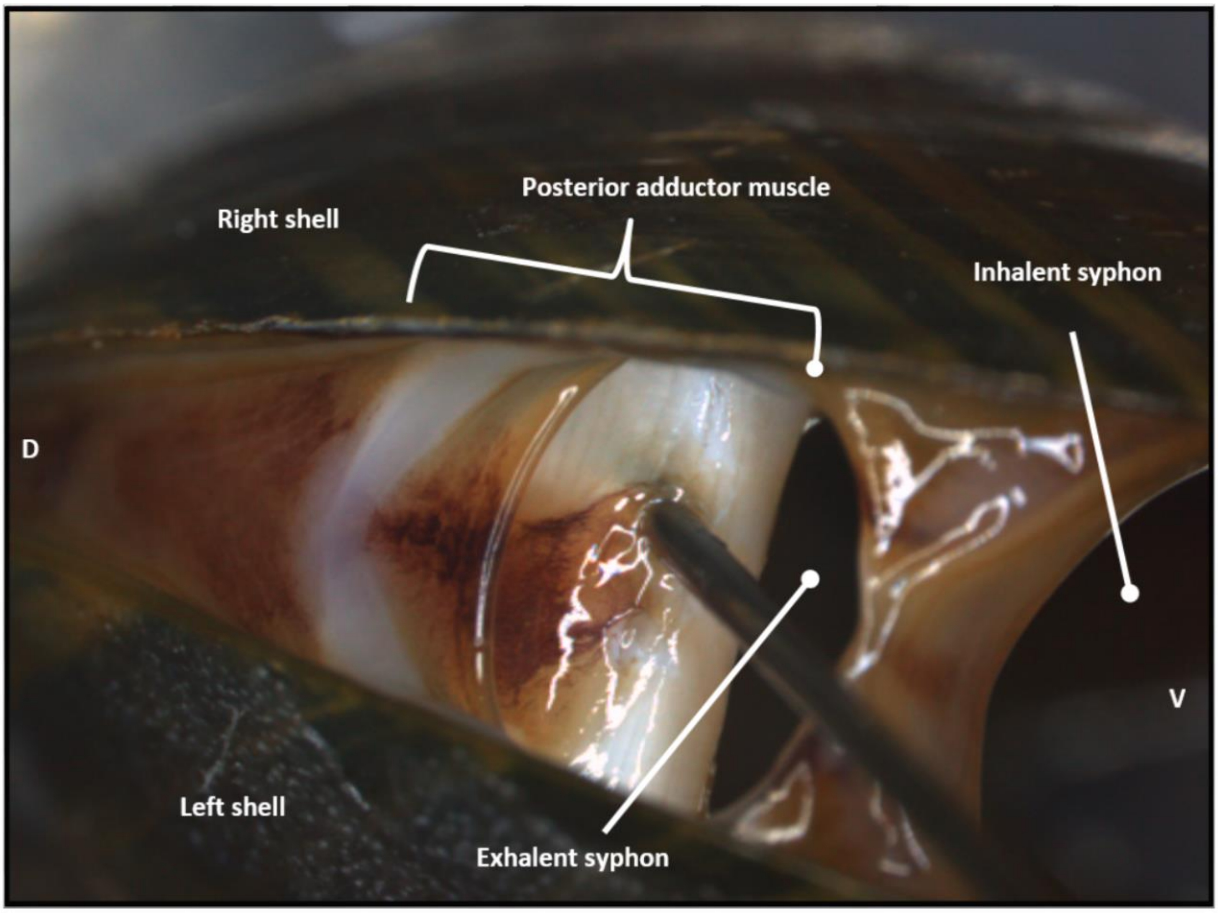

Supplementary Figure S5 : Hemolymph withdrawal from the posterior adductor muscle in the blue mussel (*Mytilus edulis)*. Abbreviations: D, Dorsal; V, Ventral.

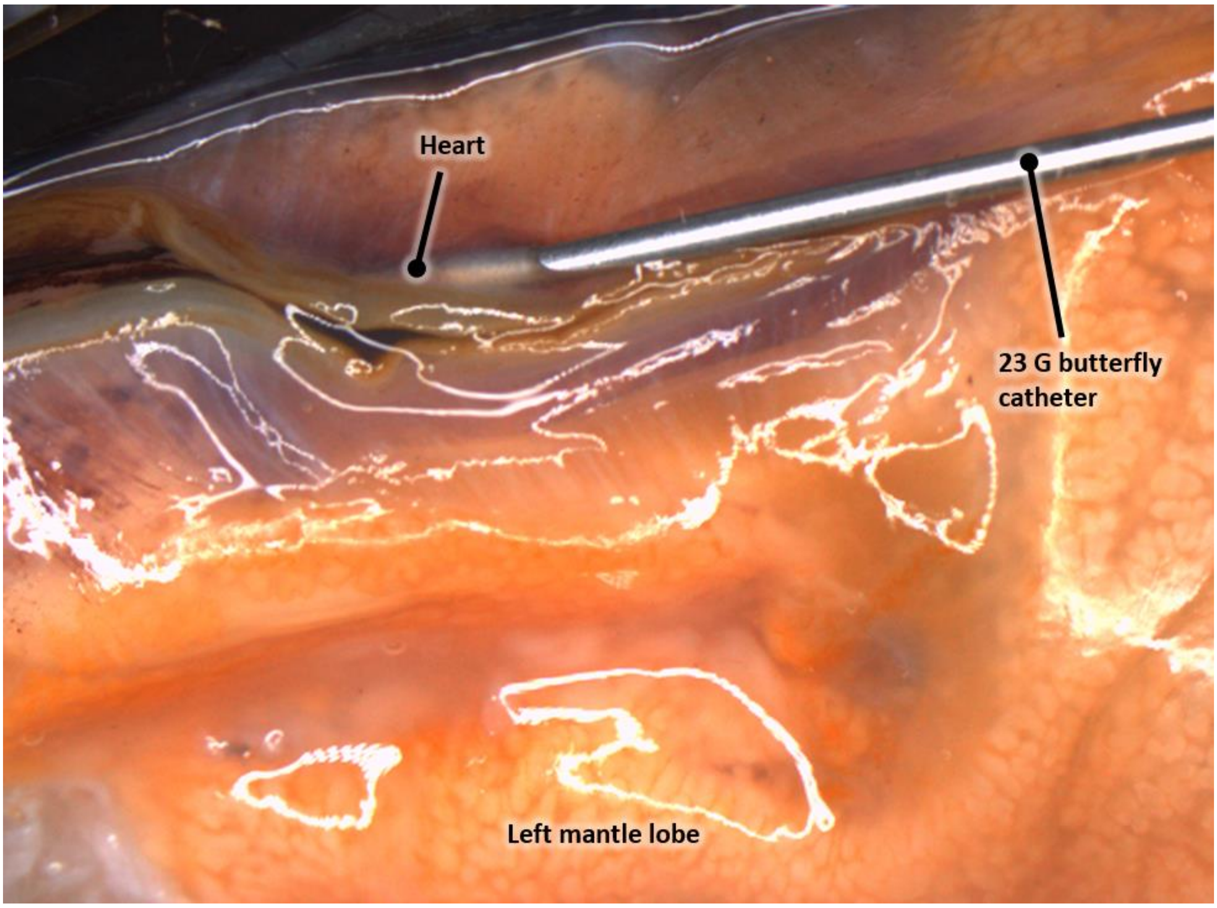

Supplementary Figure S6 : Hemolymph withdrawal from the heart in the blue mussel (*Mytilus edulis)*.

Supplementary movie legend

Supplementary Movie 1: Mytilus_edulis_anatomy_3D. A 3D-reconstruction of histological serial sections of *Mytilus edulis* visualizes the cardiovascular system (heart and related blood vessels), gastro-intestinal (GI) tract, gills, as well as the metanephridia, and the adductor and retractor muscles in relation to the foot. Colour labels: brown: labial palps, green: GI-tract, red: ventricle, orange: heart atria, bordeaux: blood vessels, purple: pericard, pink: oblique vein, dark blue: intrarenal vasculature, light blue: pericardio-renal canal, white: muscle tissue, brown: mussel foot, yellow: gills
